# Evaluation of Strategies to Separate Root-Associated Microbial Communities: A Crucial Choice in Rhizobiome Research

**DOI:** 10.3389/fmicb.2016.00773

**Published:** 2016-05-24

**Authors:** Tim Richter-Heitmann, Thilo Eickhorst, Stefan Knauth, Michael W. Friedrich, Hannes Schmidt

**Affiliations:** ^1^Microbial Ecophysiology, Faculty of Biology/Chemistry, University of BremenBremen, Germany; ^2^International Max Planck Research School for Marine Microbiology, Max Planck Institute for Marine MicrobiologyBremen, Germany; ^3^Soil Microbial Ecology, Faculty of Biology/Chemistry, University of BremenBremen, Germany

**Keywords:** rhizosphere, rhizoplane, endosphere, root microbiome, microbial abundance, sonication

## Abstract

Plants shape distinct, species-specific microbiomes in their rhizospheres. A main premise for evaluating microbial communities associated with root-soil compartments is their successful separation into the rhizosphere (soil-root interface), the rhizoplane (root surface), and the endosphere (inside roots). We evaluated different approaches (washing, sonication, and bleaching) regarding their efficiency to separate microbial cells associated with different root compartments of soil-grown rice using fluorescence microscopy and community fingerprinting of 16S rRNA genes. Vigorous washing detached 45% of the rhizoplane population compared to untreated roots. Additional sonication reduced rhizoplane-attached microorganisms by up to 78% but caused various degrees of root tissue destruction at all sonication intensities tested. Treatment with sodium hypochlorite almost completely (98%) removed rhizoplane-associated microbial cells. Community fingerprinting revealed that microbial communities obtained from untreated, washed, and sonicated roots were not statistically distinguishable. Hypochlorite-treated roots harbored communities significantly different from all other samples, likely representing true endospheric populations. Applying these procedures to other root samples (bean and clover) revealed that treatment efficiencies were strongly affected by root morphological parameters such as root hair density and rigidity of epidermis. Our findings suggest that a careful evaluation of separation strategies prior to molecular community analysis is indispensable, especially when endophytes are the subject of interest.

## Introduction

Soils host an important interface where organisms of several taxonomic kingdoms live and interact with each other: the rhizosphere. Plants influence the soil surrounding their roots by nutrient and water uptake as well as the release of rhizodeposits, gasses, and protons, consequently applying selective pressure to microorganisms (Hinsinger et al., 2009). In turn, microorganisms colonizing rhizosphere habitats may contribute to plant growth (Compant et al., 2010) and health (Berendsen et al., 2012), for example, by assisting in nutrient uptake or by suppressing plant pathogens. Especially since the advent of high-throughput sequencing techniques (Knief, 2014), research on the dynamics, functionality, and composition of the rhizosphere and its associated microbiome has become a highly relevant topic that has been extensively reviewed (e.g., Philippot et al., 2013; Berg et al., 2014; Vandenkoornhuyse et al., 2015). Rhizosphere microenvironments are frequently separated into rhizosphere soil (soil-root interface), rhizoplane (root surface), and endosphere (inner root), each possessing distinct features to which microorganisms have to adapt (McNear, 2013). Studying communities residing in these microenvironments requires reliable separation strategies. Hereby, two basic steps are most commonly carried out (Hirsch and Mauchline, 2012): (i) The rhizosphere community is obtained by manually removing bulk soil from roots, followed by washing, brushing or shaking off adhering soil particles (e.g., Marschner et al., 2004; Donn et al., 2015); (ii) the rhizoplane community is removed from the root surface by chemical or mechanical treatment and the remaining root material is then considered to contain only endophytes (e.g., Gottel et al., 2011; Edwards et al., 2015). Recently, sonication has emerged as the method of choice to detach rhizoplane or rhizosphere colonizers from the root surface using a variety of sonication devices, intensities, and pulse durations (Supplementary Table S1). Although sonication has been proven to be more effective in removing bacterial cells from their substratum than homogenizers, vortex mixers, and blending devices (Buesing and Gessner, 2002), its application as a “best-practice-protocol” for root surfaces was very recently challenged (Reinhold-Hurek et al., 2015) by comparing bacterial gene copy numbers among untreated roots and roots treated by sonication.

Other researchers have omitted sonication entirely, and instead carried out washing with or without additional shaking (Chave et al., 2008; Ofek et al., 2014), shaking only (Nunan et al., 2005), bead beating (Peiffer et al., 2013), or combined several mechanical treatments (Kaiser et al., 2001; Wieland et al., 2001). A detailed summary of protocols (not including sonication) from studies before 2009 has been compiled by Barillot et al. (2013). Another route to achieve community separation involves chemical surface sterilization, which is frequently applied in studies targeting root endophytes. Ethanol has been used for rhizoplane removal as a sole chemical agent (Bodenhausen et al., 2013) or in combination with sodium hypochlorite (NaOCl; da Silva et al., 2014). The sole application of NaOCl as a surface sterilizing agent is more common, for example, in endophyte research of rice (Reinhold et al., 1986) - either in combination with (Sessitsch et al., 2012) or without prolonged bead beating (Hardoim et al., 2011). Other example plant species, whose endosphere has been studied with the help of NaOCl bleaching, include cardon cacti (Puente et al., 2009), Portuguese broom (Becerra-Castro et al., 2011), or – in combination with H_2_O_2_ – poplar trees (Gottel et al., 2011). However, NaOCl readily reacts with nucleic acids and peptides (Prince and Andrus, 1992; Hawkins et al., 2003) and therefore can be expected to impact downstream nucleic acid analysis (Lundberg et al., 2012).

Substantial achievements in understanding root microbiomes have been made by applying a variety of separation techniques. Based on the analysis of microbial community composition of the root compartments (i.e., rhizosphere, rhizoplane, and endosphere), it now is well established that these three habitats host distinct communities in *Arabidopsis thaliana* (Bulgarelli et al., 2012; Lundberg et al., 2012; Bodenhausen et al., 2013), *Oryza sativa* (Edwards et al., 2015), or *Populus deltoides* (Gottel et al., 2011). These studies additionally reported a significant decrease in species richness within the longitudinal gradient from bulk soil to endosphere. It also appears that plant species shape their individual microbiota from bulk soil, as host specific signatures on the rhizoplane were found in wheat, maize, cucumber, and tomato (Ofek et al., 2014), and in four different tropical tree species (Oh et al., 2012). Even genotypes shaped significantly different rhizosphere communities, as demonstrated for potato (Inceoglu et al., 2011), soy bean (Xu et al., 2009), maize (Peiffer et al., 2013), and rice (Edwards et al., 2015), although this effect may change with plant age (Inceoglu et al., 2011; Chaparro et al., 2014) and was less pronounced when ecotypes of *A. thaliana* were compared to other species with different taxonomic distances (Schlaeppi et al., 2014).

Many of these studies applied different sonication treatments (amongst other techniques) to separate root-associated microbial communities followed by an interpretation of the results based on high-throughput sequencing techniques (e.g., Bulgarelli et al., 2012; Lundberg et al., 2012). However, the success of different separation strategies is not commonly assessed and is often based on agar plating as the only means to confirm the sterility of treated roots although the weaknesses of cultivation-dependent techniques for such purposes are widely accepted. To date, a systematic evaluation of such sample pre-treatments and their potential effect on downstream molecular analyses is missing. We therefore put emphasis on comparing different sonication techniques (with varying intensities and pulse durations) along with frequently used washing and bleach treatments. We systematically applied fluorescence microscopy and community fingerprinting of 16S rRNA genes to evaluate the efficacy of separation strategies with respect to the removal of rhizoplane-associated microorganisms and root surface integrity of wetland rice. Moreover, we addressed if the analysis of microbial community compositional data could be affected by selected treatments.

## Materials and Methods

### Rice Cultivation and Root Sampling

Seeds of *O. sativa* L. (cultivar IR 36) were surface sterilized in 5% NaOCl for 2 min, followed by three washes in autoclaved ultrapure water. The seeds were pre-germinated in the soil used for the experiment for 2 weeks. Individual seedlings were transplanted and cultivated on a Chinese paddy soil (silt loam; C/N: 9.0; OM: 1.2%; pH: 5.5) in three replicate pots under controlled climatic conditions (Schmidt et al., 2011). Eight weeks after transplanting, whole plants including roots and soil were removed and transferred to a laboratory tray filled with autoclaved water. The roots were gently washed free of soil, cut into segments of 2 cm in length, and immediately stored in 1 × PBS:absolute ethanol (PBS:EtOH; 2:3, v/v) at -20°C. For the following procedures, segments of 2 cm sampled at 4–6 cm from the root tip were used representing mature root tissues.

### Separation of Rhizoplane-Associated Microorganisms

Root segments remained either untreated (Treatment T1; **Table 1**) or were washed in 5 ml tubes containing 3 ml of 1 × PBS amended with 0.2% Silwet (PBS-S, Obermeier, Germany; T2) on an orbital shaker (300 rpm, 15 min, 4°C). Washed roots were then transferred to 15 ml tubes (five roots per treatment) containing 2 ml 0.2% PBS-S and subjected to sonication using a sonication probe [T3, T4; Sonopuls HD2200 equipped with probe MS73 (output frequency 20 kHz, power 200 W, amplitude: 310 μm), Bandelin, Germany] or a sonication bath with adaptive cavitation technique [T5, T6; Bioruptor Plus (output frequency 20 kHz, power 160 W), Diagenode, Belgium] with two levels of intensity each (**Table 1**). Tubes containing roots were cooled (+4°C) during sonication steps to prevent heat damage of root tissue and microbial cells. Alternatively, washed roots were treated by submerging in 2 ml 5% NaOCl for 2 min followed by three washes in autoclaved ultrapure water (T7). Washed, sonicated, and bleached roots were divided into two batches and (i) stored at -20°C for DNA extraction and (ii) stored in PBS-EtOH (2:3, v/v) at -20°C for fluorescence microscopy. The PBS-S solutions remaining in the tubes after washing and sonication treatments (treatment suspensions; T2–T6) were also divided into two batches and (i) stored at -20°C for DNA extraction and (ii) fixed in 4% formaldehyde-PBS (2.5 h, 4°C), washed in PBS, and stored in PBS-EtOH (2:3, v/v) at -20°C.

**Table 1:** Treatments used to separate microbial cells from rice rhizoplanes.

Treatment label	T1	T2	T3	T4	T5	T6	T7
Treatment procedure	Untreated	Washed (shaker)	Sonication probe^1^	Sonication probe	Sonication bath^2^	Sonication bath	Bleached
Solution	–	0.2% PBS-S	0.2% PBS-S	0.2% PBS-S	0.2% PBS-S	0.2% PBS-S	5% NaOCl in PBS
Duration	–	15 min	5 × 30 s	2 × 30 s	5 × 30 s	10 × 30 s	2 min
Intensity	–	300 rpm	10% power	40% power	Low	High	

*^1^Sonication probe, 3 mm tip (Bandelin Sonopuls HD2200 equipped with MS73). ^2^Sonication bath with adaptive cavitation technique (Diagenode Bioruptor Plus). Treatments T3–T6 were applied subsequent to washing (T2).*

### Microscopic Analysis of Rhizoplane Colonization

Root segments were transferred into a petri dish, covered with 50 μl of SYBR-Green I staining dye (10x; Lumiprobe, Germany) and incubated at room temperature for 15 min in the dark. Root segments were destained with sterile ultrapure water for 5 min and mounted in antifading medium (Vectashield H-1000, Vector Laboratories, USA). Cover slips were fixed with carbon pads which served as spacer ensuring a parallel placement of the cover slips and prevented destruction of root tissue. Microscopy was performed with a fluorescence microscope (Axio Imager M2, Zeiss, Germany) and a 63× objective (Zeiss, Germany).

Microbial cells colonizing the root segments were first observed with a double excitation filter set (F51-009, AHF, Germany) in order to prove positive staining of microorganisms on top of plant tissue-derived autofluorescent background. Greyscale images were then taken with an AxioCam MRm digital camera (Zeiss, Germany) and AxioVision 4.8 software (Zeiss, Germany). A total range of approximately 20 μm in depth (*z*-axis) was selected per image to encompass root surface topology and single layers were recorded with blue- and green excitation every 0.5 μm distance along the *z*-axis. Subsequently, greyscale images were replaced with false colors and merged to single image projections per microscopic field of view. For cell counting, eight spots were selected along the root longitudinal axis with a distance of approximately 2.5 mm. Consequently, eight image stacks were recorded and analyzed for each of the three biological replicate roots per treatment. Cells were counted by marking every stained cell in the merged z-stack images in selected regions of interest, which were defined using image analysis software (AnalySIS, SoftImaging, Germany). Respective cell numbers were referred to the area of the corresponding region of interest and presented as colonization densities (cells per mm^2^).

### Microscopic Analysis of Detached Microbial Cells

Treatment suspensions containing microbial cells, root tissue debris, and soil particles detached by washing and sonication were fixed and stored in 2 ml PBS-EtOH (see above). Aliquots of 750 μl were filtered through polycarbonate filters (0.2 μm, GTTP25, Millipore, Ireland). Filter sections were embedded in mounting medium containing the general cell stain DAPI (Vectashield H-1200, Vector Laboratories, USA) and analyzed by epifluorescence microscopy (Axioskop 2, Zeiss, Germany) using a DAPI filter set (F46-000, AHF, Germany) and a 40× objective (Zeiss, Germany). Images were taken on 20 spots for each filter section following a line from the center to the outer edge of the polycarbonate filter to account for filtration inhomogeneity. Total cells were counted in these images by marking stained cells using AnalySIS image analysis software (SoftImaging, Germany). All counting data were extrapolated to cells per root segment in the respective treatment suspension. In parallel, cells associated with fragments of root tissue or soil particles were enumerated. Moreover, the quantity and size of fragments of root tissue and soil particles was assessed.

### DNA Extraction

The DNA of all samples (roots and suspensions containing detached cells) was extracted using the NucleoSpin Soil Kit (Macherey-Nagel, Germany) with buffer SL1 and enhancer SX. Root segments were first ground in liquid nitrogen in micro reaction cups and manually comminuted with spatula. Afterward, buffer and beads were added to the bead beating tube and the extraction was finalized according to manufacturer’s instructions. DNA from detached cells was extracted after filtering the treatment suspension through polycarbonate membranes (0.2 μm, 25 mm diameter, Sartorius, Germany). The membranes were placed into the aforementioned bead beating tubes and DNA extraction was performed according to the manual. For sample lysis (physical disruption) a swing mill (MM200, Retsch, Germany) was used with 25 Hz for 30 s. The DNA of all samples was eluted in 30 μl elution buffer (5 mM Tris/HCl, pH8.5) and stored at -20°C.

### Microbial Community Analysis

The composition of microbial communities associated with roots and detached from roots was analyzed by terminal restriction fragment length polymorphism (T-RFLP). For the amplification of 16S rRNA genes the bacterial primer set 27F [FAM-5′-AGA GTT TGA TCM TGG CTC AG-3′; (Lane, 1991)] and 907R [5′-CCG TCA ATT CCT TTR AGT TT-3′; (Lane, 1991)] was used. PCR was carried out with the following cycle scheme: initial denaturation for 2 min at 94°C, followed by 30 cycles of 30 s denaturation at 94°C, 30 s annealing at 52°C, and 30 s elongation at 72°C, and a final elongation step for 7 min at 72°C. The reactions had a volume of 50 μl containing 5 μl of buffer II, 1.25 U DreamTaq polymerase and 20 μg of BSA (Fermentas, Germany). The final concentrations were 0.5 μmol l^-1^ of each primer and 50 μmol l^-1^ of each nucleotide.

Amplicons were purified with the MinElute kit (Qiagen, Germany). 120 ng of PCR product was digested with *Msp*I (cutting site C| CGG, New England Biolabs, USA), and the FAM-labeled fragments were purified using SigmaSpin Post-Reaction Clean-Up columns (Sigma–Aldrich, Germany). Three microliter of digested product were added to a mix of X-Rhodamine MapMarker 1000 (BioVentures, USA) and Hi-Di formamide (1:50; Applied Biosystems, USA). After incubation at 95°C for 5 min, the samples were analyzed with a 3130 Genetic Analyzer (Applied Biosystems, USA). Raw data was analyzed with the TREX online suite^1^. Peaks representing sequence lengths shorter than 50 base pairs or longer than 900 base pairs were discarded from the dataset. The denoising process implemented in TREX was used to identify true peaks (Abdo et al., 2006), and the final OTU matrix was assembled binning of peaks within 0.5 base pairs. Abundance data was calculated as peak heights relative to total peak height per sample. The primer set used is able to bind to the rRNA genes of plastids and plant mitochondria. *In silico* digestion with *MspI* of plant-derived DNA was performed and the influence of the plant-associated peaks on the analysis was tested. We thus removed the peak at 493 base pairs from the dataset, which was identified as the 16S rRNA gene from plastid DNA.

### Statistical Analysis

Statistical analyses of cell counts and microbial community analyses were conducted in the R environment (R Development Core Team, 2015) using the packages vegan (Oksanen et al., 2015) and multcomp (Herberich et al., 2010). For cell and particle counting experiments, significant differences between treatment outcomes were analyzed with the generalized linear hypothesis test [function glht()] after calculation of generalized linear models under the Poisson distribution and using the Tukey multiple comparison procedure adapted for high heteroscedasticity. *P*-values were adjusted for multiple testing according to the default setting of glht(). Statistical significance was assumed when adjusted *p*-values were below α = 0.05. For community profile data, OTU richness per sample was assessed by the calculation of Hill numbers *D* of order *q* = 0 and *q* = 2 based on peak height data, representing unweighted (^0^*D*, species richness) and abundance-weighted (^2^*D*, linearized Shannon diversity) richness estimates (Chao et al., 2013; Sokol et al., 2013). We performed non-metric multidimensional scaling (NMDS) of Hellinger-transformed abundance data using the Bray–Curtis distance and tested for significant influence of different treatments on sample ordination with anosim (9999 per mutations). Individual peaks appearing once in the entire dataset were removed before analysis (Culman et al., 2008).

## Results

**FIGURE 1:**
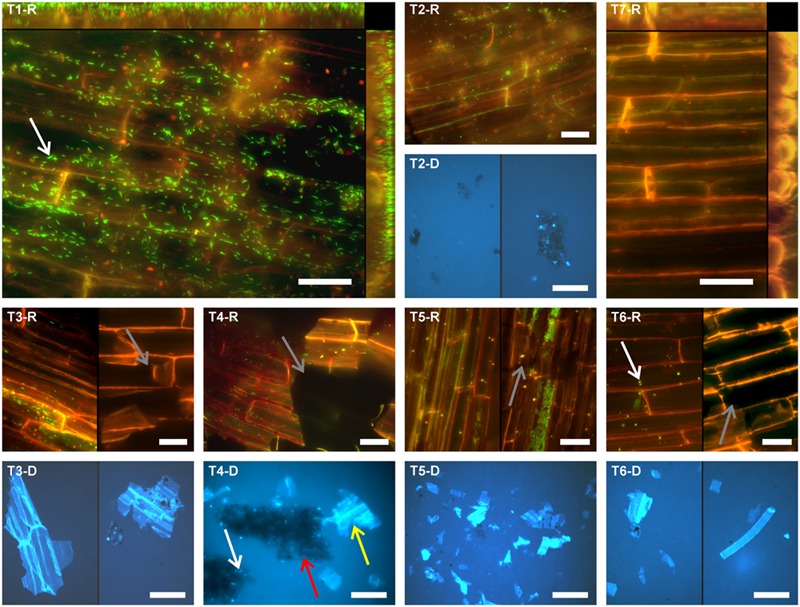
**Fluorescence micrographs showing the microbial colonization of differently treated rice root surfaces.** T1: untreated, T2: washed, T3: sonication probe (low intensity), T4: sonication probe (high intensity), T5: sonication bath (low intensity), T6: sonication bath (high intensity), T7: treated with NaOCl. For T2–T6 respective fluorescence micrographs of the detached cells and root fragments are presented (#-D). Microorganisms can be observed in green fluorescence (SYBR-Green I stain) in rhizoplane images (#-R) and bright blue fluorescence (DAPI stain) in the micrographs showing detached microbial cells (#-D), respectively. Scale bar represents 20 μm in rhizoplane images (#-R) and 50 μm in images of samples with detached material (#-D). T1-R and T7-R show merged z-profiles along both edges of the respective images. Arrows exemplarily indicate microbial cells (white), root tissue disruption (gray), soil particles (red), and root fragments (yellow).

Root segments of *O. sativa* were subjected to different treatments to separate root-associated microbial communities. Separation efficiency was evaluated by *in situ* counting of cells on root surfaces and in treatment suspensions containing detached microbial cells. Moreover, the quantity and size of root tissue debris and soil particles detached from roots and contained in treatment suspensions was assessed. Representative images of treatment outcomes are depicted in **Figure 1**.

### Microbial Cell Counts

**FIGURE 2:**
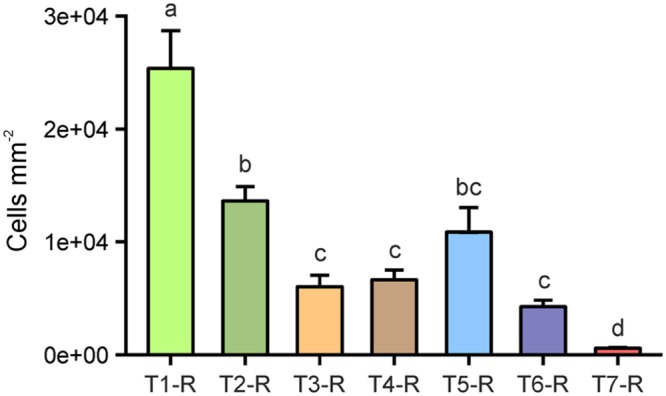
**Microbial cells enumerated on rice root surfaces (#-R) after the application of separation treatments.** T1: untreated, T2: washed, T3: sonication probe (low intensity), T4: sonication probe (high intensity), T5: sonication bath (low intensity), T6: sonication bath (high intensity), T7: treated with NaOCl. Letters indicate significant differences (*p* < 0.05); error bars: standard error (*n* = 3).

Cells counted in merged z-stack images at eight spots per replicate root segment of individual plants from all treatments were extrapolated to root surface area (mm^2^). In our assessment, an untreated rice root segment in 4–6 cm distance to the root tip hosted an average of 2.48 × 10^4^ cells per mm^2^ [±5.4 × 10^3^ standard error of the mean (SE), **Figure 2**], which also included tightly attached rhizosphere soil-associated microorganisms. Washing as a single treatment (T2) resulted in an average of 1.35 × 10^4^ cells per mm^2^, which were still attached to rice root surfaces, and accounted for a loss of 55% compared to the initial average cell count (**Figure 2**). By washing plus subsequent probe sonication (T3 and T4), the microbial cell colonization decreased by approximately 75%. The highest cell decrease was found after treatment with the sonication bath at highest energy (T6), reducing the initial colonization density by 78%. No significant differences were found in cell counts between the four sonication treatments tested, although results from probe sonication (T3 and T4) were more homogeneous between replicates than the sonication bath (T5 and T6). In total, the sonication procedures were approximately 1.7 times more effective in cleaning root surfaces from colonizing cells compared to washing alone. In contrast, the treatment with NaOCl (T7) resulted in nearly complete removal of the rhizoplane community (**Figure 1**, T7-R), leaving only 2.4% of the initial colonization attached to the rhizoplane. Increasing intensity, probe and especially bath sonication (T5 and T6) led to a significantly heterogeneous distribution of remaining cells within and between replicates (*p* < 0.05; Supplementary Figure S1).

**FIGURE 3:**
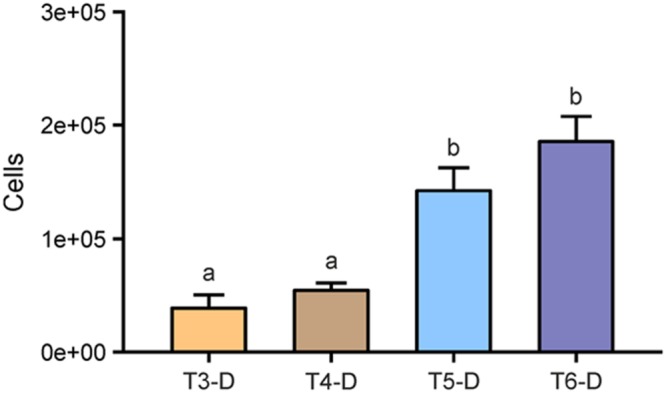
**Detached microbial cells enumerated in suspensions (#-D) after sonication treatments.** T3: sonication probe (low intensity), T4: sonication probe (high intensity), T5: sonication bath (low intensity), T6: sonication bath (high intensity). All roots have previously been subjected to washing (T2) resulting in 5.23 × 10^4^ (±1.20 × 10^4^ SE) cells detached per root segment. Letters indicate significant differences (*p* < 0.05); error bars: standard error (*n* = 3).

We also counted cells that were detached from roots during washing (T2) and subsequent sonication treatments T3–T6. Respective treatment suspensions were filtered and during enumeration, cells were differentiated between root debris-associated, soil particle-associated, and free-floating microbial cells (**Figure 3**; **Table 2**). Bath sonication lead to significantly higher numbers of detectable microbial cells in the respective suspensions compared to probe sonications (*p* < 0.05), with no effect of sonication intensity. Numbers of detached cells ranged from 3.96 × 10^4^ cells per root segment in the suspension of T3 to 1.85 × 10^5^ cells of T5 (**Figure 3**), in addition to an average of 5.23 × 10^4^ cells already detached during the washing step. Hence, the sonication bath treatments removed 272 and 355% more stainable cells from the rhizoplane with low and high energy, respectively, in comparison to the washing step alone. Similar to the observation on rhizoplanes, no detectable signal was observed in filtered suspensions obtained from NaOCl-treated roots indicating the dissociation of double-stranded DNA (Hawkins et al., 2003). In general, we found less variance in cell counts of treatment suspension replicates, as compared to total cell counts of the root surface.

**Table 2:** Analysis of detached rhizosphere soil particles, root tissue debris, and associated cell numbers in suspensions after washing (T2) and subsequent sonication treatments: T3: sonication probe (low intensity), T4: sonication probe (high intensity), T5: sonication bath (low intensity), T6: sonication bath (high intensity).

Treatment		T2	T3^∗^	T4^∗^	T5^∗^	T6^∗^
Cells associated with detached fragments per root segment	Soil particles	2.2E+04	8.5E+03	8.6E+03	1.6E+03	2.2E+03
		±8.7E+03	±4.9E+03	±3.4E+03	±9.9E+02	±1.6E+03
	Root tissue	5.1E+03	1.2E+04	2.1E+04	2.0E+04	1.2E+04
		±5.1E+03	±6.7E+03	±4.7E+03	±9.7E+03	±3.2E+03

Number of detached fragments per root segment	Soil particles	4.6E+04	2.2E+04	2.3E+04	2.2E+04	2.8E+04
		±1.8E+04	±8.8E+03	±1.2E+04	±8.1E+03	±1.0E+04
	Root tissue	3.0E+03	1.2E+04	2.4E+04	6.2E+04	3.2E+04
		±1.6E+03	±8.6E+03	±3.3E+03	±2.1E+04	±2.5E+03

Average size of detached fragments (μm^2^)	Soil particles	125	92	226	114	107
		±49	±19	±86	±65	±27
	Root tissue	346	443	639	263	163
		±133	±248	±143	±98	±32

*^∗^Sonication was applied subsequent to washing (T2). Numbers are given as mean of *n* = 3 individual roots. ±: standard error.*

While evaluating suspensions of the individual treatments, we recognized microbial cells associated with either root tissue fragments or soil particles. Thus, we further compared the abundance of these cells still attached to soil or root particles after the respective separation treatments, which were in general lower than free-floating microbial cells (**Table 2**). Suspensions from roots subjected to the washing procedure (T2) featured more cells attached to soil (42% of all detached cells) than to root debris (10% of all detached cells), indicating a well-preserved structure of soil particles removed from the rhizoplane via washing. This effect was reversed when samples were sonicated subsequently to washing. Thus, the proportion of soil associated cells decreased by more than 60% in the suspensions of the sonication probe treatments (T3 and T4) and 90% in the respective samples of the sonication bath treatment (T5 and T6) compared to washing (T2). By contrast, a higher number of cells in the suspensions of the sonication probe treatments were still attached to root fragments accounting for 29 and 39% of all detached cells in T3 and T4, respectively. Following treatments T5 and T6, only very few cells could be observed being associated to either fraction of root tissue (around 10%) or soil particles (1%; **Table 2**) although the total number of detached cells had increased in this treatment (**Figure 3**).

### Root Tissue Destruction

Root tissue disruption was commonly observed after sonication, as evident from rhizodermis imaging and root tissue debris in treatment suspensions (**Figure 1**, T4-R, T3-D–T5-D). All sonication methods were able to compromise rhizoplane integrity (**Figure 1**, T3R–T6-R). However, a clear difference was observed among the two sonication techniques as clear cuts and losses of larger fragments were obvious for probe sonication (T3 and T4). Bath sonication (T5 and T6) resulted in disruption of tissue within root cell walls and smaller fragments in the respective suspensions. We assessed these microscopic observations quantitatively by determining the abundance and size of root fragments in treatment suspensions (**Table 2**). Respective counts were higher in the suspensions of sonication bath methods, especially when compared to the washing treatment itself (T2) which was almost devoid of root debris. High intensity probe and bath sonication procedures caused comparable rhizoplane damage, while low intensity sonication bath procedures showed the strongest effect, with high variance within replicates. However, both sonication bath methods tended to produce smaller root tissue fragments compared to the other methods.

### Removal of Soil Particles from Root Surfaces

For identifying true rhizoplane colonizing microorganisms, the absence of any rhizosphere-related contaminations (i.e., soil particles with microorganisms) is required. In our experiments, all treatment suspensions containing detached microbial cells featured soil particles of different sizes showing the removal of rhizosphere soil from the rhizoplane (**Table 2**; **Figure 1**, T4-D). The highest proportion of soil particles was removed from the root surface after washing (T2). Sonication resulted in the detachment of additional 47–59% soil particles compared to washing alone, while no significant differences between sonication protocols were observed. Aggregates of soil particles detached via the washing treatment (**Figure 1**, T2-D) and probe sonication (**Figure 1**, T4-D) were observed to be larger compared to soil particles detached via bath sonication. Soil particles still adhering to the rhizoplane were frequently found after sonication with both low and high intensity (Supplementary Figure S2).

### Microbial Community Fingerprinting

In order to assess biological richness and diversity in root samples and treatment suspensions containing detached microorganisms, T-RFLP analyses of 16S rRNA genes were performed. A total of 255 individual peaks in 36 samples (root samples T1–T7 and post-treatment suspensions T2–T6, all in triplicates) were observed after removal of singletons and the putative plastid peak (TRF 493 bp). Root samples treated with NaOCl had the lowest average OTU richness ^0^*D* (14 ± 6 SE), whereas communities detached from roots showed the highest average richness (67 ± 20–105 ± 11). Untreated roots (T1) featured high OTU richness (86 ± 12). However, as evident from the high standard errors of the mean, the OTU richness differed highly between replicates, suggesting that observed β-diversity between root communities is not always the result of the treatment effect. Thus, the inversed Simpson concentration index (^2^*D*) was calculated for each community, which puts more emphasis on changes in the abundances of the dominant OTUs. For root samples, a steady decrease in ^2^*D* values was found between untreated roots and NaOCl-treated roots with washed and sonicated root samples ranking in between, except T6 (^2^*D*_T1-R_: 7.3 ± 1.6 SE; ^2^*D*_T2-R_: 4.3 ± 0.5; ^2^*D*_T3-R_: 3.2 ± 0.3; ^2^*D*_T4-R_: 3.9 ± 0.5; ^2^*D*_T5-R_: 4.4 ± 0.6; ^2^*D*_T6-R_: 7.3 ± 0.6; ^2^*D*_T7-R_: 2.1 ± 0.2). For the root samples, ^2^*D* values were higher for the sonication bath treatments than for the probe sonicated roots. For microbial assemblies detached from roots, much higher values for ^2^*D* were observed in general, and no clear trend was visible due to high within-sample heterogeneity (^2^*D*_T2-D_: 11.0 ± 3.1 SE; ^2^*D*_T3-D_: 12.1 ± 1.8 ^2^*D*_T4-D_: 8.8 ± 0.5; ^2^*D*_T5-D_: 7.6 ± 0.7; ^2^*D*_T6-D_: 10.0 ± 3.7).

**FIGURE 4:**
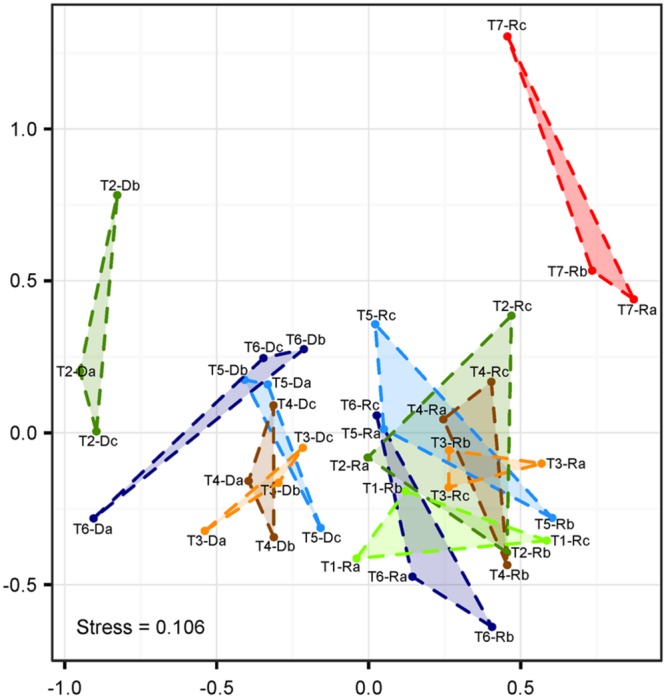
**Non-metric multidimensional scaling (NMDS) of T-RFLP fingerprints (Hellinger-transformed abundance data).** Labeling represents roots (#-R) and root-detached microorganisms (#-D) for different treatments (T1–T7), and replicates (*n* = 3 each, a–c). T1: untreated, T2: washed, T3: sonication probe (low intensity), T4: sonication probe (high intensity), T5: sonication bath (low intensity), T6: sonication bath (high intensity), T7: treated with NaOCl.

Non-metric multi-dimensional scaling based on Bray–Curtis-distances revealed a clear separation of populations sampled from remaining roots and treatment suspensions along the first axis (**Figure 4**). The highest distance was observed between communities in the suspensions with detached cells obtained from the washing step (samples T2-D) and root-associated communities after NaOCl-treatment most likely representing the endophytic community (samples T7-R). The NMDS analysis was complemented with anosim under 999 permutations, which showed a significant (*p* < 0.001) difference of the community structures between treatments. Within the ordination space, root samples (including untreated roots) were undistinguishable from root samples obtained after washing and sonication (T1-R–T6-R). Only root-associated microbiota from samples incubated in NaOCl clustered on their own.

## Discussion

Our understanding of the dynamics of root-associated microbial communities rapidly improves by utilizing high-throughput sequencing techniques (Knief, 2014). However, our current definitions of the different root-associated communities are constrained by technical limitations (i.e., incomplete microbiome separation). Implications of these limitations on root microbiome research need to be assessed, especially when molecular tools are used that are able to detect very small subpopulations (i.e., rare biosphere). In this study our aim was to evaluate the efficacy of different techniques that are commonly used in plant microbiome research.

### Root Washing is Insufficient for Endosphere Studies

It is often assumed that rhizosphere soil can be removed from the root surface by washing alone (Knief et al., 2012; Turner et al., 2013). In this study, we rigidly washed roots in a detergent-amended buffer while shaking for 15 min. The microscopic observation revealed that considerable amounts of soil were still attached to roots after the washing treatment (Supplementary Figure S2), which was further corroborated by the presence of considerable amounts of soil particles in sample buffers obtained after subsequent sonication steps. Thus, washing alone may have accidentally included rhizosphere-associated populations in a number of studies when analyzing microbial communities in the rhizoplane and/or endophytic compartments. Nevertheless, washing of roots did not compromise root surface integrity in our study, and thus, may represent the best approach for including the analysis of rhizoplane-colonizing cells *in situ* (e.g., via fluorescence and scanning electron microscopy).

### Sonication Fails to Completely Remove Rhizoplane Colonizers

Compared to washing treatment, subsequent sonication resulted in additional detachment of cells (approximately 30%) from the rhizoplane but failed to completely remove microbial cells thereof. These findings obtained via quantification of single microbial cells were supported by NMDS ordination of T-RFLP data. Bacterial communities in untreated, washed and sonicated roots remained indistinguishable (**Figure 4**), which further indicated that no bacterial groups were preferentially detached from the rhizoplane. Although fingerprinting methods such as T-RFLP may not reach the analytical resolution of high-throughput sequencing, they are well suited to detect changes in β-diversity (van Dorst et al., 2014). Thus, our findings obtained via T-RFLP analysis may be carefully used to interpret results of root microbiome studies based on high-throughput sequencing. If sonication is the method of choice, our results show neither probe nor bath methods were fully effective in removing rhizoplane-associated populations. In our assessment, the choice of the instrument played a negligible role. If the detachment of intact cells to the treatment suspension is considered important (e.g., for cultivation or DNA based methods), sonication bath devices were found to be slightly superior to probe sonication. These findings should be taken into account when sonication is selected for the analysis of endophytic microorganisms in roots. In a similar vein, the analysis of phyllosphere endophytes may also be affected by incomplete removal of surface-associated microorganisms via sonication (e.g., Bodenhausen et al., 2013; Bai et al., 2015). However, this has to be evaluated separately.

### Root Destruction May Affect Community Analysis

Regardless of the technique used, sonication seriously compromised the integrity of the outer root layer (**Figure 1**). The problem of root disruption for downstream analysis is twofold: firstly, root endophytes may be incorporated into the rhizoplane sample and bias its community composition. However, based on T-RFLP analysis, root tissue disruption by sonication was not detected as the composition of communities of detached cells (T3-R–T6-R) was different from that of other treatments (T3-D–T6-D; **Figure 4**). Secondly, sonication triggers the loss of cells colonizing outer root layers, and thus, leads to potential changes in the endophytic community (e.g., reduced α-diversity). The latter may apply to a number of studies, where sonication was performed in addition to washing and where scanning electron microscopy revealed the immense level of root tissue destruction (Bulgarelli et al., 2012; Lundberg et al., 2012; Lebeis et al., 2015). Microbial assemblages of sonicated roots clearly separated from communities detached thereof (**Figure 4**). The application of a standardized sonication protocol should presumably lead to a sufficient separation into rhizoplane and endorhizosphere communities as demonstrated for a range of plant species (for example Gottel et al., 2011; Edwards et al., 2015). However, none of the sonication treatments tested was successful in entirely removing rhizoplane-associated communities from roots (**Figure 2**). Consequently, it remains difficult to interpret whether observed differences in microbial communities of previous studies are blurred by technical limitations of the separation protocol, especially when appropriate controls are not comparatively evaluated (e.g., NaOCl-treated and untreated roots). Such studies would then rather describe the detachable fraction of rhizoplane-colonizing microorganisms and an endosphere compartment that is contaminated by cells resisting sonication. This methodological bias has to be considered, especially when sonication is applied to address research questions investigating mechanisms involved in the selection of endophytes by roots.

In a recent study on root-associated microbiomes of rice, Edwards et al. (2015) applied three cycles of probe sonication (30 s each, output frequency 42 kHz, power 90 W), and surprisingly, they were not able to detect bacteria colonizing rice rhizoplanes while the root tissue appeared rather undamaged. Consequently, the authors concluded that the complete removal of rhizoplane-associated microorganisms had been successfully achieved via probe sonication. In contrast, we frequently observed root tissue disruption after probe sonication at even lower intensity (T3) while using five cycles (30 s each, output frequency 20 kHz, power 20 W) and still detected microbial cells colonizing the rhizoplane in every replicate sample. The low technical reproducibility observed with just slightly different treatment protocols for the same plant underlines the importance of evaluating and adapting separation techniques even for different cultivars of the same plant species. However, it remains difficult to compare the results of Edwards et al. (2015) to our findings as they used CARD-FISH for monitoring purposes, which relies on the coverage and hybridization success of an oligonucleotide probe (here EUB338) to a specific group of microorganisms. To ensure that all archaea and bacteria are indeed included, researchers aiming to confirm a successful removal of rhizoplane-colonizing microorganisms should consider the presented SYBR-Green staining as a quick and reliable method to detect microbial cells *in situ*. Another recent study reported that the sonication of roots (*O. sativa* and *A. thaliana*) in a water bath for 15 min did not result in a decrease of rhizoplane-colonizing bacteria as assessed by quantification of 23S rRNA gene copy numbers (Reinhold-Hurek et al., 2015). Technical information on the sonication treatment (e.g., type of instrument, intensity, and volume) were not disclosed rendering the comparison to our findings difficult. In general, we recommend that future publications opting for sonication to separate rhizoplane-associated microorganisms should state the necessary details of the sonication device to ensure reproducibility, as these details are frequently lacking (Supplementary Table S1). Nevertheless, Reinhold-Hurek et al. (2015) concluded (i) that sonication may not always represent the method of choice and (ii) that surface sterilization of roots by chemical treatment may be best suited when the endophytic compartment should be investigated.

### Bleaching Facilitates Recovery of Endophytes

Treating root samples with NaOCl was the only strategy to remove almost all rhizoplane-colonizing cells (97.6%) while retaining the integrity of the outer cell layer. Despite the high variability of diversity within treatments [also reported by Oh et al. (2012)], only the communities of NaOCl-treated roots were statistically separated from untreated, washed, and sonicated roots, most probably representing the “true” endophytic community. This view is supported by the fivefold decrease in inverse Shannon diversity in NaOCl-treated roots, which is in agreement with previous reports of decreasing species richness between the ecto- and endorhizosphere (Bulgarelli et al., 2012; Lundberg et al., 2012; Ofek et al., 2014). However, the treatment of root samples with surface sterilizing agents such as NaOCl has shortcomings. NaOCl is known to degrade nucleic acids (Prince and Andrus, 1992; Hawkins et al., 2003), and thus, may penetrate into root tissue and destroy microbial and plant DNA. Nevertheless, we recommend to chemically sterilize the root surface carefully before nucleic acid extraction if endophytes are the focus of research. Ethanol could serve as an alternative (Bodenhausen et al., 2013) but was not tested in our study. In any case, the compound of choice should be evaluated for its potential to penetrate root tissues and incubation times should be adjusted to minimize the loss of viable cells in root interiors (Jha et al., 2009).

### Implications of Root Colonization and Morphology

We observed a high variability of microbial cell numbers among biological replicate root segments. This was already pronounced in untreated samples, and thus, not related to individual separation treatments, which indicates that different roots of individual plants have varying success in recruiting microbial populations from soil. Especially on the surface of relatively young roots (our study: 10 weeks), highly irregular colonization patterns can be expected (Schmidt and Eickhorst, 2014). The apparent variability in root-colonizing populations needs to be considered when roots of young plants are used for comparative community analysis. Moreover, the high variability among roots of individual plants asks for further replication efforts or homogenization of sufficiently large samples in future studies on root microbiomes.

As a final note, our findings seem to be highly dependent on the plant species (i.e., morphological root parameters). Applying the tested protocols to roots of *Vicia faba* and *Trifolium pratense* led to different surface cleaning efficacies (Supplementary Figure S3), as we found even higher variability among replicates and between treatments. *V. faba* roots, being comparable in diameter to *O. sativa* roots, featured massive amounts of root hairs that interfered with all treatments. The dense net of root hairs (Supplementary Figure S3, T5-V) seemed to protect rhizoplane-associated cells from being detached by washing and sonication, while “unprotected” areas of low root hair density featured tissue disruption similar to rice roots (Supplementary Figure S3, T4-V). Again, incubation of root segments with NaOCl was the only treatment to almost completely remove rhizoplane colonizing microorganisms. Roots of *T. pratense* are considerably finer than roots of *V. faba* and *O. sativa*, and thus highly sensitive to sonication procedures. Even after low intensity sonication a strong effect on root tissue integrity was commonly observed, which made the quantification of cell numbers nearly impossible. High intensity sonication occasionally resulted in a complete disruption of root segments. These observations strongly suggest that protocols for the separation of root-associated microbial communities should be adapted and tested in advance for the plant species of interest. As a consequence of different responses of root tissue to separation strategies, the comparison of microbial communities associated with different plant species (e.g., Schlaeppi et al., 2014; Bulgarelli et al., 2015) should be interpreted carefully when the same washing and sonication protocol is used for roots of different morphology.

## Conclusion

The selection of a separation strategy with respect to plant microbiome analysis remains a critical issue and has to be adapted to the respective research questions. Probe and bath sonication may not always represent a strategy that reliably removes microorganisms and soil particles from roots and has the potential to severely compromise root tissue integrity. Future studies that apply sonication should clearly state the necessary details of the sonication device to ensure comparability among studies. Washing of roots may represent the best approach when researchers aim to include the analysis of rhizoplane-colonizing cells *in situ*. Studies focusing on endophytes should carefully apply chemical agents such as NaOCl to remove rhizoplane-associated cells from the root surface. We recommend appropriate replication and rigorous testing of selected separation strategies in respect to reproducibility among replicates and species. The presented approach using SYBR-Green-based fluorescence microscopy represents a prime choice for quick and easy control of separation efficiency.

## Author Contributions

TR-H, HS, and TE conducted and interpreted the experiments and wrote the manuscript. SK provided molecular analyses. MF was involved in the conception of the experiments and in writing of the manuscript.

## Conflict of Interest Statement

The authors declare that the research was conducted in the absence of any commercial or financial relationships that could be construed as a potential conflict of interest.

## Footnotes

1 http://trex.biohpc.org/
