# Supplementary material for: Evaluation of Strategies to Separate Root-Associated Microbial Communities: A Crucial Choice in Rhizobiome Research
Source: Front Microbiol. 2016 May 24;7:773. doi: 10.3389/fmicb.2016.00773 (PMC4877504; doi:10.3389/fmicb.2016.00773)
Supplement: Supplementary file 1 [file Data_Sheet_1.PDF]

Supplementary Material

Evaluation of Strategies to Separate Root-Associated Microbial Communities: A Crucial Choice in Rhizobiome Research

Tim Richter-Heitmann, Thilo Eickhorst, Stefan Knauth, Michael W. Friedrich and Hannes Schmidt*

***Correspondence:** *Hannes Schmidt h.schmidt@uni-bremen.de*

Supplementary Figures

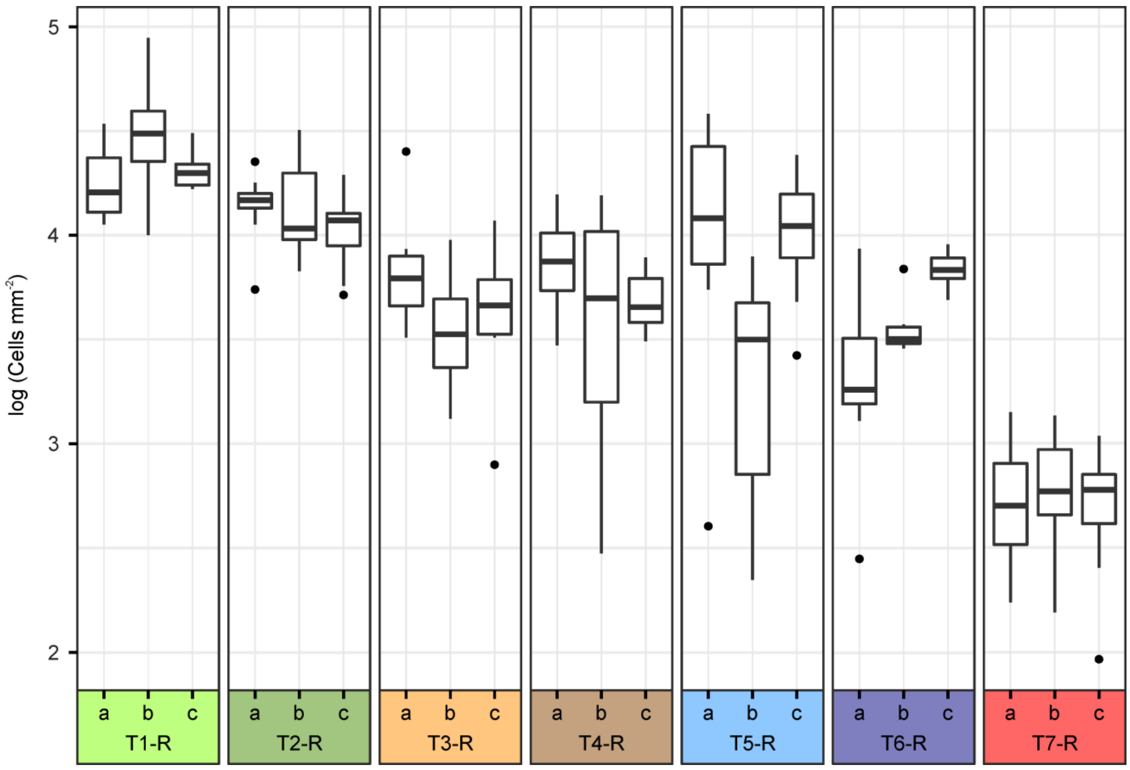

Supplementary FIGURE 1 │ Root surface-associated cell counts (log-scaled per mm²) for seven different treatments. T1: untreated, T2: washed, T3: sonication probe (low intensity), T4: sonication probe (high intensity), T5: sonication bath (low intensity), T6: sonication bath (high intensity), T7: treated with NaOCl. Depicted are boxplots of 8 observations per biological replicate (n = 3; letters a, b, c).

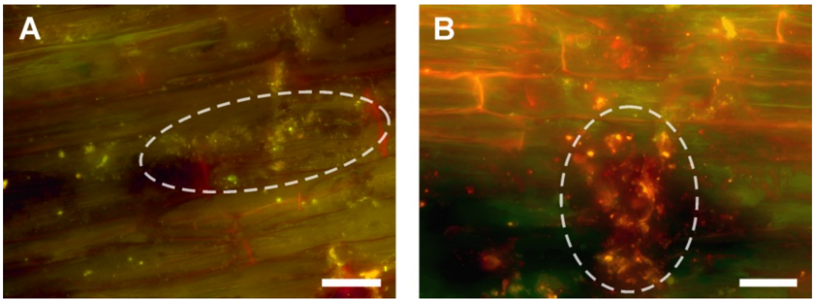

Supplementary FIGURE 2 │ Z-stack projections of fluorescent images of rice roots after treatment with sonication bath. A: low intensity; B: high intensity. Circles show soil particles still attached to the rhizoplane after sonication. Microorganisms can be observed in green fluorescence (SYBR-Green I stain). Scale bar: 20 µm.

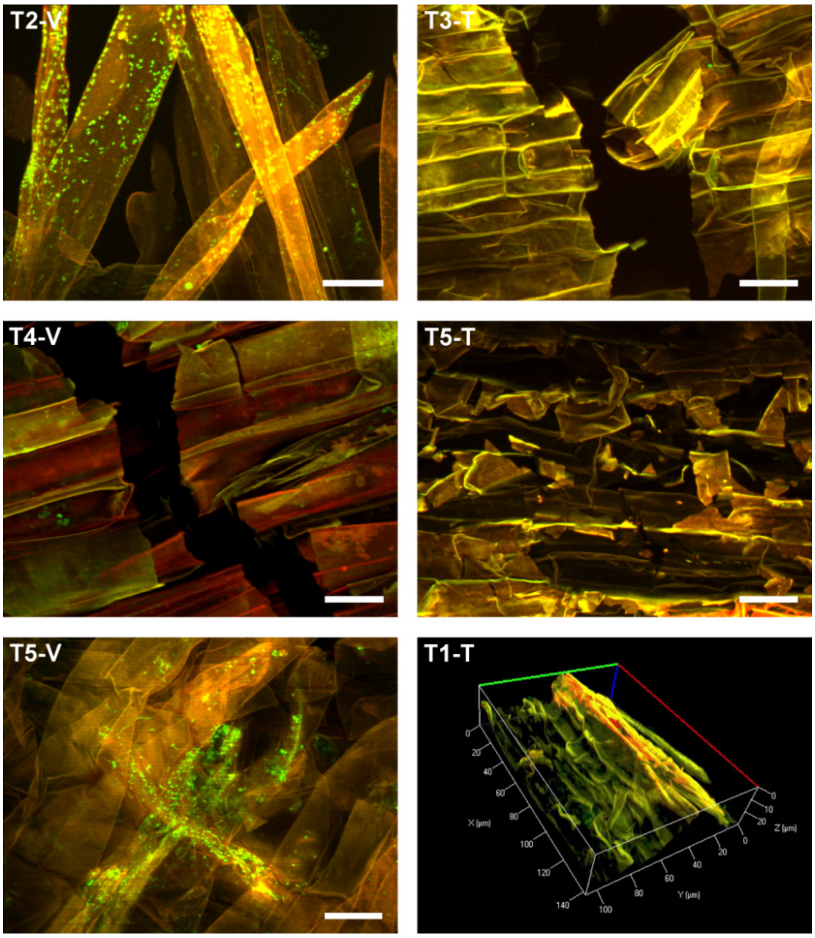

Supplementary FIGURE 3 │ Selected fluorescence micrographs of roots of *Vicia faba* (#-V) and *Trifolium pratense* (#-T) after separation treatments. T2: washed, T3: sonication probe (low intensity), T4: sonication probe (high intensity), T5: sonication bath (low intensity). T1-T: 3D-projection of an untreated root of *Trifolium pratense*. Microorganisms can be observed in green fluorescence (SYBR-Green I stain). Scale bar: 20 µm.

Supplementary Tables

Supplementary TABLE 1 │ Selected sonication protocols for roots and their applications as found in a literature survey.

Sonicator	Protocol	Target species	Target habitat	Reference
Ultrasonic probe<br>Branson Sonifier	4 min (output control 2, power and frequency n/a); brief pauses every minute	*Oryza sativa*	Root surface biofilm	Briones *et al.* (2002)
Ultrasonic probe<br>Branson 510	5 × 1 min<br>(135 W, frequency n/a) and vortex shaking (1 min)	*Dulichium arundinaceum*<br>*Sarracenia purpurea*	Rhizoplane	Cadillo-Quiroz *et al.* (2010)
Ultrasonic probe<br>Branson, unspecified	Rhizoplane: 1 × 30 s<br>(90 W, 42 kHz)<br>Endosphere: 3 × 30 s<br>(90 W, 42 kHz)	*Oryza sativa*	Endosphere<br>Rhizoplane	Edwards *et al.* (2015)
Ultrasonic bath<br>Thermo Fisher Scientific FS20	3 × 60 s<br>(70 W, 42 kHz)	*Glycine max*	Different layers of the Ectorhizosphere	White *et al.* (2015a, b)
Ultrasonic bath with adaptive cavitation technique<br>Diagenode Bioruptor UCD-300	10 × 30 s<br>(160 W, frequency n/a);<br>30 s pauses	*Arabidopsis thaliana*<br>*Hordeum vulgare*	“Root compartment”	Bulgarelli *et al.* (2012)<br>Bulgarelli *et al.* (2015)
Ultrasonic bath with adaptive cavitation technique<br>Diagenode Bioruptor	5 × 30 s<br>(power n/a, low frequency);<br>30 s pauses	*Arabidopsis thaliana*	“Endophytic compartment”	Lundberg *et al.* (2012)<br>Lebeis *et al.* (2015)
Ultrasonic bath<br>Manufacturer n/a	15 min<br>(power and frequency n/a)	*Arabidopsis thaliana*<br>*Oryza sativa*	Endosphere	Reinhold-Hurek *et al.* (2015)
Ultrasonic washer<br>Manufacturer n/a	2 × 3 min<br>(power and frequency n/a)	*Oryza sativa*	Rhizosphere	Doi *et al.* (2011)
n/a	"shaking, vortexing, and sonicating every 30 s … with alternations for 3 min"<br>(power and frequency n/a)	*Agathis borneensis,*<br>*Dipterocarpus kerrii*<br>*Dyera costulata*<br>*Gnetum gnemon*	Rhizoplane	Oh *et al.* (2012)
n/a	6 × 1 min<br>(power and frequency n/a);<br>5 min pauses; additional 10 s vortex shaking	*Arabidopsis thaliana*	Endo- and Ecto-rhizosphere	Bodenhausen *et al.* (2013)

References Supplementary TABLE 1

Briones, A. M., Okabe, S., Umemiya, Y., Ramsing, N.-B., Reichardt, W., and Okuyama, H. (2002). Influence of Different Cultivars on Populations of Ammonia-Oxidizing Bacteria in the Root Environment of Rice. *Appl. Environ. Microbiol.* 68, 3067–3075. doi: 10.1128/aem.68.6.3067-3075.2002

Cadillo-Quiroz, H., Yavitt, J. B., Zinder, S. H., and Thies, J. E. (2010). Diversity and community structure of Archaea inhabiting the rhizoplane of two contrasting plants from an acidic bog. *Microb. Ecol.* 59, 757–767. doi: 10.1007/s00248-009-9628-3

Edwards, J., Johnson, C., Santos-Medellin, C., Lurie, E., Podishetty, N. K., Bhatnagar, S., et al. (2015). Structure, variation, and assembly of the rootassociated microbiomes of rice. *Proc. Natl. Acad. Sci. U.S.A.* 112, E911–E920. doi: 10.1073/pnas.1414592112

White, L. J., Brözel, V. S., and Subramanian, S. (2015a). Isolation of rhizosphere bacterial communities from soil. *Bio Protoc.* 5:e1569.

White, L. J., Jothibasu, K., Reese, R. N., Brozel, V. S., and Subramanian, S. (2015b). Spatio temporal influence of isoflavonoids on bacterial diversity in the soybean rhizosphere. *Mol. PlantMicrobe Interact.* 28, 22–29. doi: 10.1094/MPMI-08-14-0247-R

Bulgarelli, D., Garrido-Oter, R., Münch, P. C., Weiman, A., Dröge, J., Pan, Y., et al. (2015). Structure and function of the bacterial root microbiota in wild and domesticated barley. *Cell Host Microbe* 17, 392–403. doi: 10.1016/j.chom.2015.01.011

Bulgarelli, D., Rott, M., Schlaeppi, K., Ver Loren van Themaat, E., Ahmadinejad, N., Assenza, F., et al. (2012). Revealing structure and assembly cues for *Arabidopsis* root-inhabiting bacterial microbiota. *Nature* 488, 91–95. doi: 10.1038/nature11336

Reinhold-Hurek, B., Bunger, W., Burbano, C. S., Sabale, M., and Hurek, T. (2015). Roots shaping their microbiome: global hotspots for microbial activity. *Annu. Rev. Phytopathol.* 53, 403–424. doi: 10.1146/annurev-phyto-082712-102342

Doi, T., Abe, J., Shiotsu, F., and Morita, S. (2011). Study on rhizosphere bacterial community in lowland rice grown with organic fertilizers by using PCR-denaturing gradient gel electrophoresis. *Plant Root* 5, 5–16. doi: 10.3117/plantroot.5.5

Oh, Y. M., Kim, M., Lee-Cruz, L., Lai-Hoe, A., Go, R., Ainuddin, N., et al. (2012). Distinctive bacterial communities in the rhizoplane of four tropical tree species. *Microb. Ecol.* 64, 1018–1027. doi: 10.1007/s00248-012-0082-2

Bodenhausen, N., Horton, M. W., and Bergelson, J. (2013). Bacterial communities associated with the leaves and the roots of *Arabidopsis thaliana*. *PLoS ONE* 8:e56329. doi: 10.1371/journal.pone.0056329
